# Supplementary figures and images for: Potential Antioxidant and Neuroprotective Effect of Polysaccharide Isolated from Digüeñe Cyttaria espinosae
Source: J Fungi (Basel). 2025 Aug 29;11(9):637. doi: 10.3390/jof11090637 (PMC12470887; doi:10.3390/jof11090637)

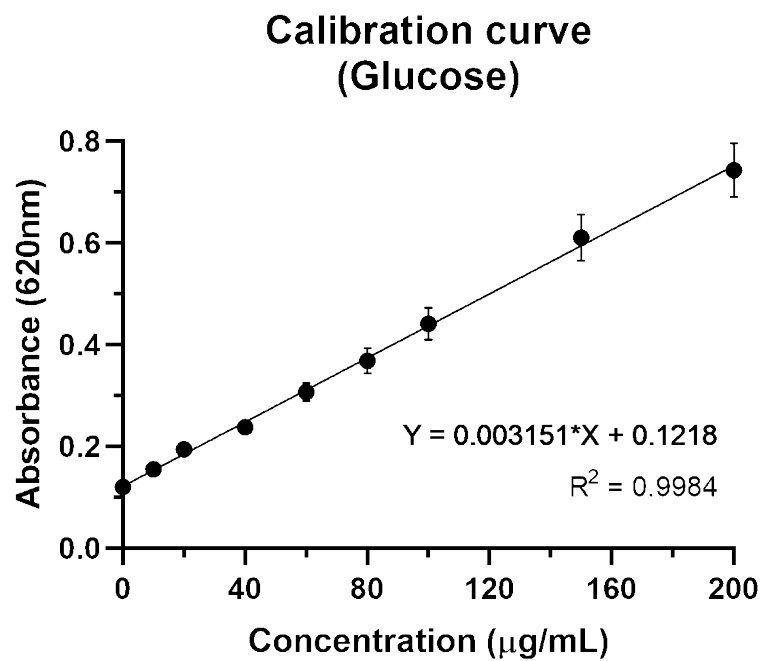

**Figure S1.** Glucose calibration curve.

Supplement: Supplementary file 1 [file jof-11-00637-s001.zip › jof-3723030-supplementary.pdf]
